# Knee articular cartilage injury treatment with matrix-induced autologous chondrocyte implantation (MACI): correlation at 24 and 120 months between clinical and radiological findings using MR arthrography

**DOI:** 10.1007/s00256-021-03775-y

**Published:** 2021-04-15

**Authors:** Marco Calvi, Marco Curti, Christian Ossola, Marta Duvia, Maria Gloria Angeretti, Mario Ronga, Eugenio Annibale Genovese

**Affiliations:** 1 Department of Radiology, University of Insubria, Varese, Italy; 2 Department of Radiology, Ospedale di Circolo, Varese, Italy; 3 Department of Orthopaedic, Messina University, Messina, Italy; 4 Intermedica-Columbus Clinical Medical Center, Milan, Italy

**Keywords:** Cartilage, Articular, Arthrography, Magnetic resonance imaging, Chondrocytes, Follow-up studies

## Abstract

**Objective:**

To evaluate the long-term evolution of matrix-induced autologous chondrocyte implantation (MACI) with magnetic resonance (MR) arthrography and verify the correlation between radiological and clinical findings.

**Materials and methods:**

Twenty-six patients (20 m/6f) were diagnosed with knee chondral injuries and treated with MACI implantation. Each patient received MR arthrography and clinical examination at mid-term (range 22–36 months) and long term (range 96–194 months) after surgery. MR arthrography was performed with dedicated coil and a 1.5-Tesla MR unit. The modified MOCART scale was used to evaluate the status of chondral implants. Implant coating, integration to the border zone, and the surface and structure of the repaired tissue were evaluated. Presence of bone marrow oedema was evaluated. The Cincinnati Knee Rating System (CKRS) was used for clinical assessment.

**Results:**

At long term, 4/26 patients had complete alignment; 5/26 had a complete integration of the margins; in 4/26 cases, the implant surface was undamaged; in 14/26 cases, the reparative tissue was homogeneous. In 9/26 cases, the implant showed isointense signal compared to articular cartilage, while the presence of subchondral bone oedema was documented in 19/26 cases. The average radiological score decreased from 59.2 (mid-term) to 38.6 (long term). The average clinical score decreased from 8.9 to 8.3.

**Conclusions:**

Decrease in clinical results was not significant (0.6 points *p* = .06), but mMOCART scores decreased significantly (*p* = .00003). Although imaging studies showed deterioration of the grafts, the patients did not have significant clinical deterioration (231/250).

## Introduction

Autologous chondrocyte implantation (ACI) was first described in 1994 and is still a surgical technique utilized to repair cartilage defects [1]. The matrix-induced autologous chondrocyte implantation (MACI) was born as an evolution of the ACI technique to simplify the surgical procedure; it is carried out in mini-arthrotomy or arthroscopy, with a significant reduction in operating times, resulting in a decrease of postoperative morbidity and the graft hypertrophy [2, 3]. This technique consists in the use of a porcine collagen membrane with seeded autologous cultured chondrocytes and involves two steps: the first step is harvesting and culturing the chondrocytes from a non-weight-bearing surface, and then seeding the chondrocytes on the collagen matrix. In the second step, the collagen matrix with the seeded chondrocytes is implanted into the osteochondral defect with fibrin glue [4].

Among the techniques described in the literature for MACI follow-up (FU) (clinical scores, arthroscopic biopsies), magnetic resonance imaging (MRI) represents an accurate and non-invasive method for the evaluation of joint cartilage [5, 6] in the post-operative period. Moreover, MR arthrography (MRa) represents the gold standard for the study of the operated knee [7] and is the most accurate method to evaluate articular cartilage injuries, increase the diagnostic reliability in the evaluation of chondral lesions and identifies the presence of any postoperative complications [8].

To our knowledge, no study in literature comprehends clinical and MRa assessment of MACI with a 10-year follow-up [8–19]: only in our previous experience in 2009, an MRa based study had a 5-year follow-up [20].

This study aims to evaluate the MRa imaging findings of MACI grafts of the knee at mid-term (range 22–36 months) and long term (range 96–194 months) after surgery and to test the correlation with the paired clinical scores.

## Materials and methods

### Ethics board review

All procedures performed in this study involving human participants were in accordance with the ethical standards of the institutional and/or national research committee and with the 1964 Declaration of Helsinki and its later amendments or comparable ethical standards. Approval for this specific study was obtained by the local institutional review board, according to the National Policy in the matter of Privacy Act on retrospective analysis of anonymized data; informed consent, as stated by *Legge 22 Dicembre 2017 n.219 Gazzetta Ufficiale della Repubblica Italiana*, was signed by each patient.

### Patient cohort

Subjects were enrolled from January 2009 to May 2019 as consecutive patients. Forty patients (24 male and 16 female average age: 30 years; mean: 28; range: 17–53) were enrolled at Ospedale di Circolo di Varese. The study was performed prospectively.

Inclusion and exclusion criteria are listed in Table 1.

**Table 1:** Inclusion and exclusion criteria

**Inclusion criteria:**	
• Age between 16 and 55 years. • Grade III to IV lesions, according to the International Cartilage Repair Society scale (ICRS). • Single or multiple lesions, even “kissing lesions” if they need the same treatment. • Lesion size greater than 1.5 cm^2^.	
**Exclusion criteria:**	
• Ongoing or past infections of the joint. • Lesions larger than 4 cm^2^. • Advanced arthrosis. • Femoral-tibial and femoral-patellar axial deviations. • Joint instability. • BMI > 30. • Ongoing infectious diseases. • Metabolic or immune system diseases. • Previous or concomitant neoplasms. • Mental retardation/mental illness that prevents a regular rehabilitation program or subsequent checks.	

### Surgical technique

All patients were treated with MACI.

Each patient was treated for a single chondral lesion except for 2 patients who were treated at the same time for both patellar and condylar lesions. Twenty-six chondral injuries involving the knee in various points were treated. Eighteen patients had previous surgery on the involved joint.

### MRa technique

MR arthrograms were performed at mid-term (mid-term follow-up; range: 22–36 months) and long term (long-term follow-up; range 96–194 months).

All MRa studies were performed with a dedicated coil (quad knee) using a 1.5-T tomograph: Eclipse, Picker-Marconi (unit 1) and Avanto, Siemens (unit 2).

MRa was performed after intra-articular injection of 40 ml of paramagnetic contrast agent (Magnevist 2 mmol/l, Bayer-Schering; Dotarem 2.5 mmol/l, Guerbet). The “unit 1” MR protocol involved the use of sagittal, coronal, or axial SE T1 sequences (TR: 500 ms, TE: 12 ms, matrix: 256 × 512, number of signal averages [NSA]: 1, thickness: 3.5–4 mm); sagittal or coronal fat-saturated FSE PD/T2 sequences (TR:4000 ms, TE: 19/96 ms, flip angle [FA]: 90°, matrix: 256 × 256, NSA: 2, thickness: 4 mm); and 3D FSE fat-saturated T1 sequences (TR: 31 ms, TE: 7 ms, FA: 90°, matrix: 256 × 256, NSA: 2, thickness: 0.50 mm) in axial or coronal planes. The “unit 2” protocol involved 3D isotropic T1 sequences (TR:16.20 ms, TE: 3.72 ms, FA: 8°, matrix: 320 × 307, NSA: 1, thickness: 0.54 mm) in the axial plane; sagittal and coronal TSE T1 sequences (TR: 667 ms, TE: 8.8 ms, FA: 143°, matrix: 384 × 307, NSA: 1, thickness: 3.5–4 mm); and sagittal or coronal FSE PD/T2 fat-saturated (TR: 3700 ms, TE: 21/125 ms, FA: 150°, matrix: 230 × 256, NSA: 1, thickness: 3.5–4 mm). Written informed consent was obtained from all patients before procedure.

### Image evaluation

The status of MACI implants was assessed using a modified version of the Magnetic Resonance Observation of Cartilage Repair Tissue (MOCART) [20], introduced by Marlovits et al. in 2004 [21].

The modified MOCART (mMOCART) we used (Table 2) did not consider post-surgical adhesions, as they resulted of minor significance in our previous experience, as well as the state of the subchondral lamina, since it is of doubtful clinical significance [20].

**Table 2:** Modified-MOCART (Magnetic resonance Observation of Cartilage Repair Tissue) Score from Genovese E. “Matrix-induced autologous chondrocyte implantation of the knee: mid-term and long-term follow-up by MR arthrography” Skeletal Radiol (2011) 40:47–56

A) Filling of defect (degree of defect repair and filling of the defect).	Complete (on a level with adjacent cartilage). Hypertrophy (over the level of the adjacent cartilage). Incomplete <50% of the adjacent cartilage. Incomplete >50% of the adjacent cartilage. Incomplete. Subchondral bone exposed (complete delamination or dislocation and/or loose body).	20 15 10 5 0
B) Integration to Context: border zone.	Complete (complete integration with adjacent cartilage). Incomplete. Demarcating border visible (split-like). Incomplete with visible defect <50% of the length of the repair tissue. Incomplete with visible defect >50% of the length of the repair tissue.	15 10 5 0
C) Surface of the repair tissue.	Surface intact. Surface damaged <50% of repair tissue depth. Surface damaged >50% of repair tissue depth.	10 5 0
D) Structure of the repair tissue.	Homogeneous. Inhomogeneous or cleft formation and/or enhancement.	5 0
E) Signal intensity of the repair tissue (dual T2-FSE fat-suppression).	Isointense. Moderately hyperintense. Markedly hyperintense.	15 5 0
F) Subchondral bone.	Normal signal. Signal intensity modification (oedema, granulation tissue, cysts, sclerosis).	5 0

Each MRa evaluation was carried out by a qualified Radiologist (EAG) with more than 20 years of experience in MRa interpretation.

**Fig. 1:**
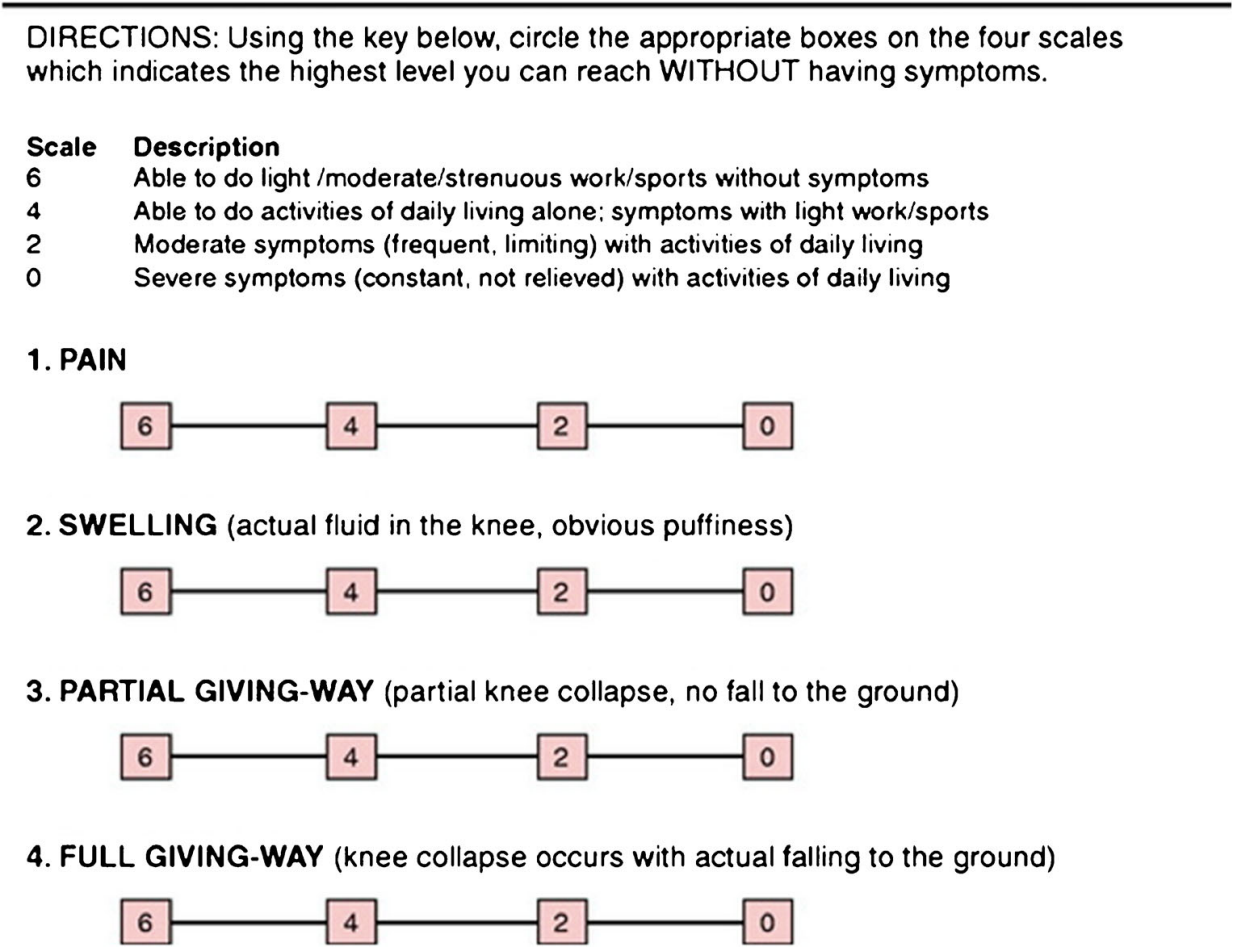
Cincinnati Knee Rating System Diagram. It represents an accurate rating of knee symptoms on a four-level scale rating scales assessing pain, swelling, partial giving way, and full giving way based on the patient perception of the overall condition of the knee. This scale was firstly proposed by Barber-Westin S. D.; Noyes, F. R.; McCloskey, J. W.: Rigorous statistical reliability, validity, and responsiveness testing of the Cincinnati Knee Rating System in 350 subjects with uninjured, injured, or anterior cruciate ligament-reconstructed knees. Am J Sports Med 27:402–416, 1999

All clinical evaluations were performed, before the MR arthrography, by the same orthopaedic surgeon (MR) with more than 20 years of clinical experience using the Cincinnati Knee Rating Scale (CKRS) [21] (Fig. 1).

The difference between the average mMOCART and the CKRS at mid-term and long-term follow-up was evaluated.

## Statistical analysis

Continuous data were tested for normality and presented as mean (± standard deviation; SD). Categorical data were presented as frequency (%).

To compare mean differences (without adjusting for confounders) between each of the categories within the mMOCART and the CKRS scores at mid-term and long-term FU, Student *t* test was conducted if assumptions were met, and non-parametric analyses (Sign test) otherwise. The results were accepted as significant at *p* < .05.

The data analysis for this paper was generated using the Real Statistics Resource Pack software (Release 7.2). Copyright (2013–2020) Charles Zaiontz. www.real-statistics.com.

## Results

**Fig. 2:**
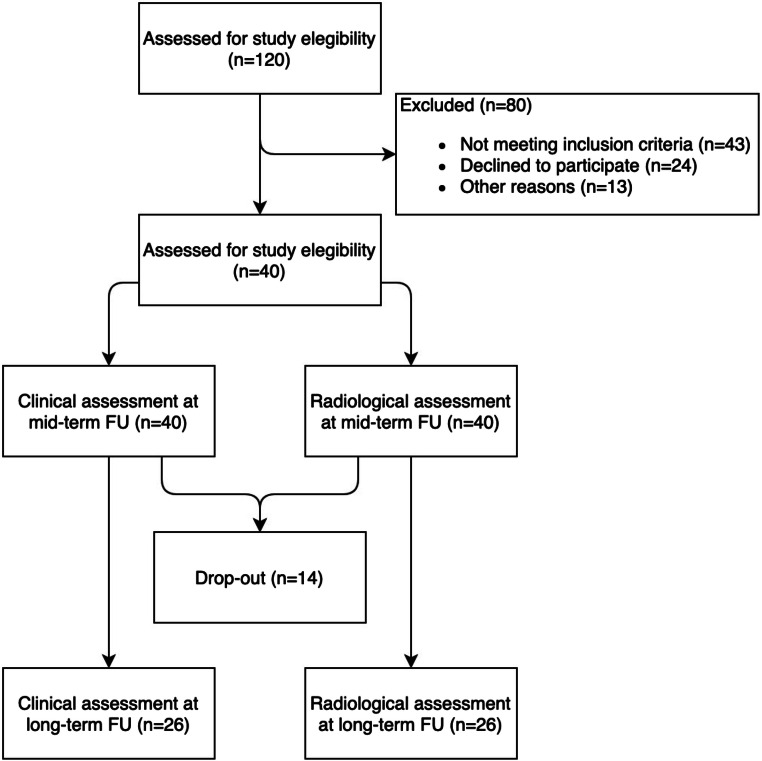
Flow-chart selection of patients in the study

**Fig. 3:**
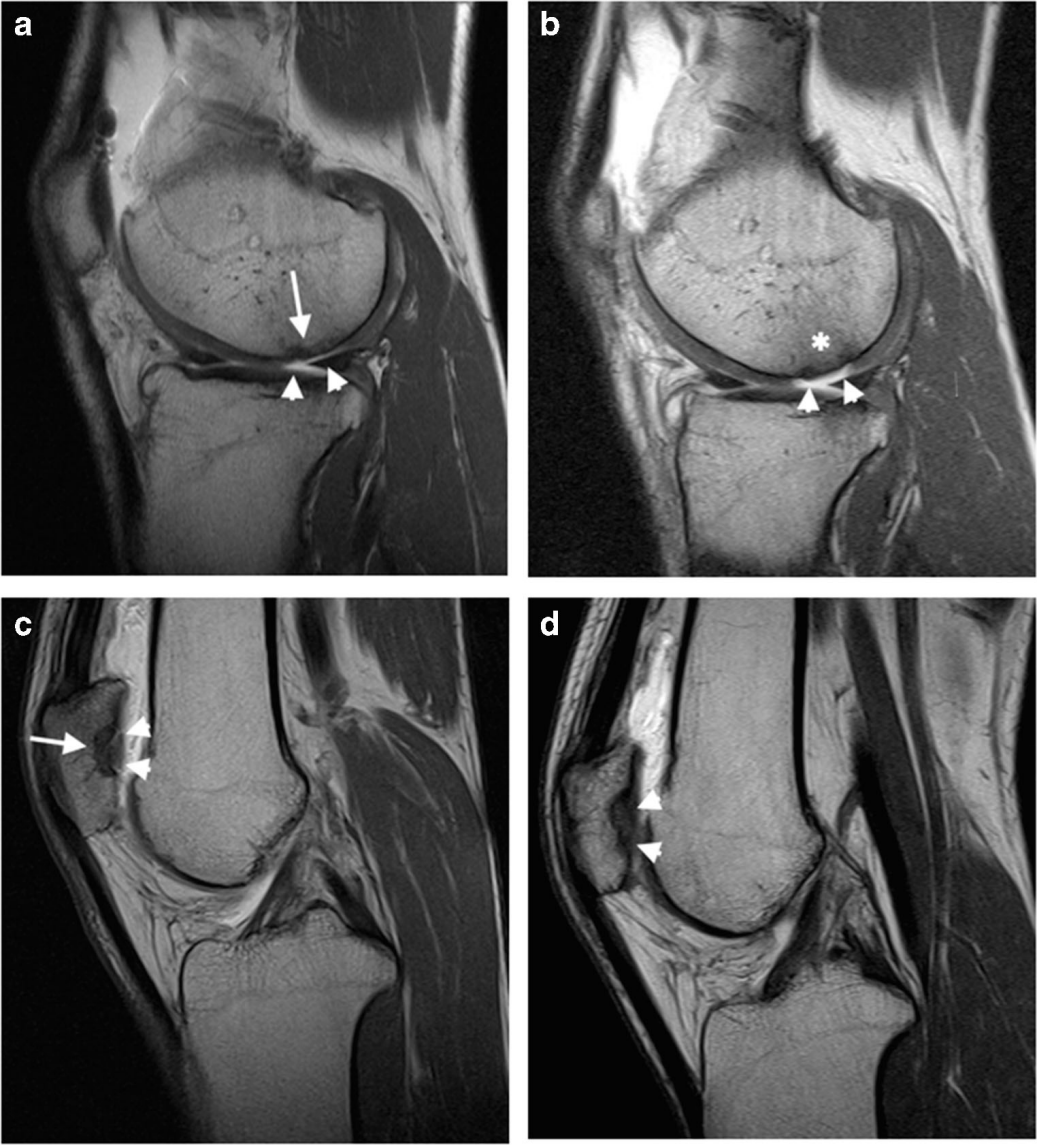
**a** Sagittal SE T1-weighted image acquired after intra articular administration of paramagnetic contrast media at mid-term. The MACI implant is evident at the posterior aspect of the internal femoral condyle (arrow). It obtained a MOCART score of 50 and a Cincinnati score of 9. The cartilage filling is incomplete, <50% of the adjacent cartilage (arrowheads). **b** The same patient repeated the exam at long term with the same parameters. The image shows deterioration of the MACI implant with a MOCART score of 40 and a Cincinnati score of 8. Subchondral bone is, in fact, exposed (arrowheads) with trabecular bone signal intensity modification compatible with bone marrow oedema (asterisk). **c** Sagittal SE T1-weighted image acquired after intra articular administration of paramagnetic contrast media at mid-term. The MACI implant is evident at the lateral patellar articular facet (arrow). It obtained a MOCART score of 50 and a Cincinnati score of 9. The cartilage filling is complete (arrowheads). **d** The same patient repeated the exam at long term with the same parameters. The implant showed good stability with a MOCART score of 60 and a Cincinnati score 9. At long term, the cartilage filling remained complete, without radiological signs of MACI degeneration (arrowheads)

Patients’ demographics and characteristics are summarized in Table 3. The patients’ average age at the time of surgery was 32.8 years. Fourteen patients refused to perform a radiological check at long-term through MRa, as they reported full subjective well-being. Twenty-six patients completed both clinical and MRI examination at 5- and 10-years follow-up (Figs. 2–3).

**Table 3:** Demographic characteristics of the sample

**Demographic characteristics**	
Patients number (*n*) Age at surgery, mean ± SD (range) (year) Sex, male/female (*n*) Follow-up time, mean ± SD (range) (year)	40 28 ± 8.9 (17–53) 24/16 8 ± 1.93 (3–11)
**Previous surgical procedures,** ***n***	18
Perforation (*n*) Meniscectomy, medial/lateral (*n*) Microfracture (*n*) ACL reconstruction (*n*) PCL reconstruction (*n*) Osteosynthesis, femoral condyle/patella (*n*)	7 3/2 3 2 0 1/0
**Defect characteristics**	
Side, right/left (*n*) Defect size, mean ± SD (range) (cm^2^)	27/15 (2 patients had concomitant patellar and condylar lesion) 4.40 ± 3.19 (130–1125)
**Defect localization**	
Medial femoral condyle Lateral femoral condyle Trochlea Patella	20 8 4 10

The MRa findings are summarised in Table 4.

**Table 4:** MRa findings at mid-term and long-term FU

Parameter	Score	No. of patients	
		Mid-term follow-up	Long-term follow-up
Filling of defect (A)	20	16	4
	15	3	2
	10	5	8
	5	2	8
	0	0	4
Integration to context: border zone (B)	15	17	5
	10	2	7
	5	6	4
	0	1	10
Surface of the repair tissue (C)	10	16	4
	5	7	12
	0	3	10
Structure of the repair tissue (D)	5	18	14
	0	8	12
Signal intensity of the repair tissue (dual T2-FSE fat-suppression) (E)	15	19	9
	10	5	14
	5	2	3
Subchondral bone (F)	5	10	7
	0	16	19

### Lining/filling of the defect (A)

**Fig. 4:**
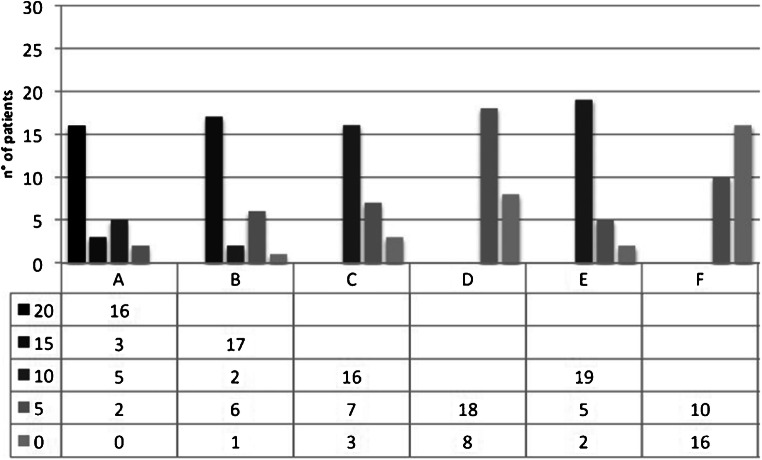
Distribution of modified mMOCART subgroup scores at mid-term follow-up (range: 22–36 months). A, B, C, D, E and F represent the categories analysed in the mMocart classification and are listed in Table 2. (A) Filling of defect (degree of defect repair and filling of the defect). (B) Integration to context: border zone. (C) Surface of the repair tissue. (D) Structure of the repair tissue. (E) Signal intensity of the repair tissue (dual T2-FSE Fat-Suppression). (F) Subchondral bone

At the mid-term FU, a complete alignment was found in 16/26 cases (61.5%); in 3/26 cases (11.5%), there was hypertrophy of the implant and in 5/26 cases (19.2%) the filling was incomplete with implant thickness greater than 50% compared to the adjacent cartilage (Fig. 4).

**Fig. 5:**
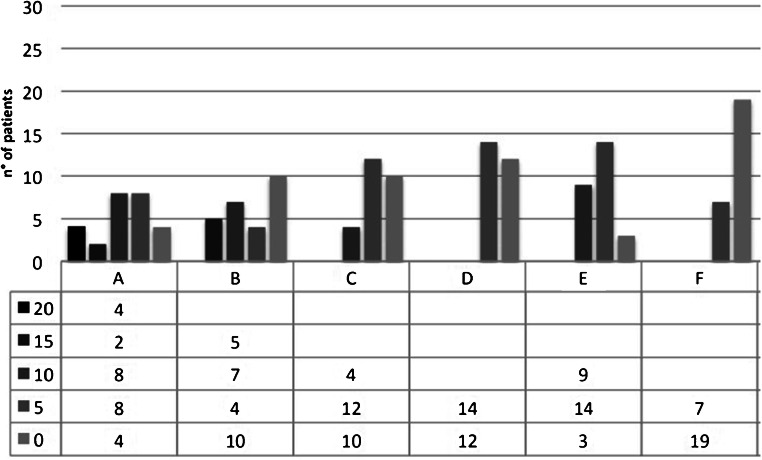
Distribution of modified mMOCART subgroup scores at long-term follow-up; range 96–194 months). A, B, C, D, E and F represent the categories analysed in the mMocart classification and are listed in Table 2. (A) Filling of defect (degree of defect repair and filling of the defect). (B) Integration to context: border zone. (C) Surface of the repair tissue. (D) Structure of the repair tissue. (E) Signal intensity of the repair tissue (dual T2-FSE Fat-Suppression). (F) Subchondral bone

At the long-term FU in only 4 cases (15.3%), the complete alignment was confirmed, while in 8 cases (30.7%) hypertrophy of the implant occurred, and in 8 cases (30.7%) the thickness was less than 50%. In long-term FU, in 4/26 cases (15.3%), the filling was incomplete and with subchondral bone exposure (Fig. 5).

### Integration to border zone (B)

At the mid-term FU in 17 cases (65.3%), there was a complete integration between the implant and native cartilage; in 2 cases (7.6%), there was incomplete integration with the visible linear interface and in 6 cases (23%) incomplete integration with a visible defect less than 50% of the implant length.

At the long-term FU in 5 cases (19.2%) it was confirmed the complete integration between native cartilage and implant, while in 7 cases (26.9%) an incomplete integration was suggested by the presence of a visible linear interface and in 10/26 cases (38.4%) it was highlighted the presence of a visible defect greater than 50% of the implant length.

### Surface of the repaired tissue (C)

At mid-term FU in 16 cases (61.5%), the implant surface was completely undamaged while in 7/26 cases (26.9%) the implant showed superficial erosion involving less than 50% of its thickness.

In the long-term FU only in 4 cases (15.3%), the implant surface was completely intact, in 12 cases (46.1%) the implant surface was damaged with thickness less than 50% of the depth of the repair tissue and in 10 cases (38.4%) there was damage with thickness greater than 50% of the depth of the repair tissue.

### Structure of the repaired tissue (D)

In the mid-term FU, homogeneity of the structure of the reparative tissue was documented in 18 cases (69.2%), while in 8/26 cases (30.7%) the structure appeared uneven.

At long-term FU, 14 of 18 maintained the same score while 4 had score deterioration.

### Signal intensity of the repaired tissue (dual T2-FSE sequences) (E)

At mid-term FU, in 19 cases (73%), isointense signal was detected. In 5 cases (19.2%), the implant appeared moderately hyperintense and in 2 cases (7.6%) there was a marked signal hyperintensity of the regeneration tissue.

At long-term FU, in 9 cases (34.6%), isointense signal was found while in 14 cases (53.8%) the implant appeared moderately hyperintense.

### Subchondral bone (F)

At mid-term FU in 10 cases (38.4%), there was complete integrity of the subchondral bone and in 16 cases (61.5%) it was present subchondral oedema.

At long-term FU, 3 patients out of 10 with bone integrity at mid-term developed subchondral oedema.

The clinical results are summarised in Table 5.

**Table 5:** Comparison of clinical and radiological findings

Patient	Mid-term Follow-up		Long-term follow-up	
	Modified MOCART score	Cincinnati score	Modified MOCART score	Cincinnati score
1	70	8	20	5
2	40	9	35	9
3	70	9	10	8
4	75	8	40	8
5	50	9	40	8
6	70	9	30	8
7	75	9	65	7
8	70	9	15	9
9	70	9	25	9
10	65	9	60	9
11	65	9	70	9
12	65	10	50	10
13	35	7	25	3
14	40	8	60	9
15	50	9	45	9
16	55	9	55	9
17	70	10	20	8
18	25	10	10	9
19	75	9	55	9
20	35	6	20	6
21	60	9	25	10
22	75	10	75	10
23	75	10	45	9
24	65	10	60	10
25	70	9	40	9
26	25	9	10	8
Average value	59.2	8.9	38.6	8.3

Clinical scores decreased in 10/26 cases (38%). Eight of 26 cases had a slight score reduction (max 2 points) while in 2/26 cases the score decreased more severely respectively from 8 to 5 points and from 7 to 3 points.

In 14/26 cases (%), the scores remained unchanged over time.

Only 2/26 patients achieved long-term FU score improvement. The average value of clinical scores decreased from 8.9 (SD ± 0.04 - mid-term FU) to 8.3 (SD ± 0.05—long-term FU).

The difference was not statistically significant (0.6 points *p* = .06).

Differences between radiological and clinical data are listed in Table 5.

Modified mMOCART scores from mid- to long-term FU almost always decreased (22/26 cases—84%); in 2/26 cases (7%), they remained unchanged and in 2/26 cases (7%) they increased.

The average value of mMOCART scores decreased from 59.2 (SD ± 16.2—mid-term FU) to 38.6 (SD ± 19.8—long-term FU). The difference was statistically significant (*p* = .00003).

Five of 26 patients obtained an average mMOCART score of 19 at the long-term follow-up, with an average clinical score of 8.8. In this subgroup, the dissociation between the two rating scales was not present at mid-term FU (average mMOCART = 68, average Cincinnati score = 9.2) (Fig. 5).

**Fig. 6:**
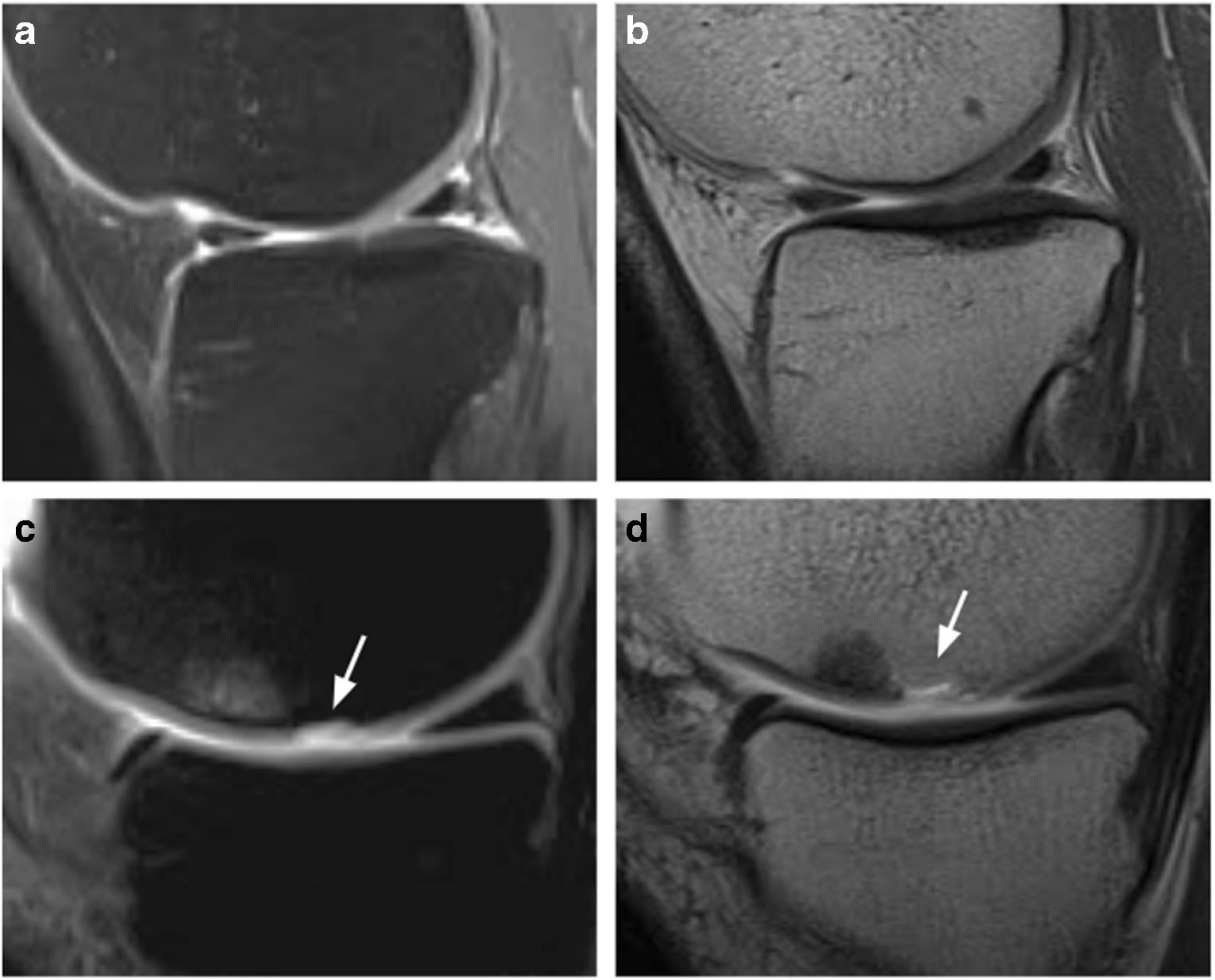
**a**, **b** A 39-year-old man treated with MACI of the internal tibial plate at mid-term with mMOCART score of 70 and Cincinnati score of 9. **a** Sagittal oblique FSE PD fat-saturated image shows non-structural incongruence of the chondral surface. **b** Sagittal oblique SE T1-weighted image. No contrast enhancement. **c**, **d** A 29-year-old woman treated with MACI of the internal femoral condyle at mid-term with mMOCART score of 25, Cincinnati score 9. **c** Sagittal oblique FSE PD fat-saturated image shows structural incongruence of the chondral surface (arrow) and subchondral bone oedema. **d** Sagittal oblique SE T1-weighted image. Contrast enhancement in the subchondral bone (arrow) [images obtained with “Unit1”]

**Fig. 7:**
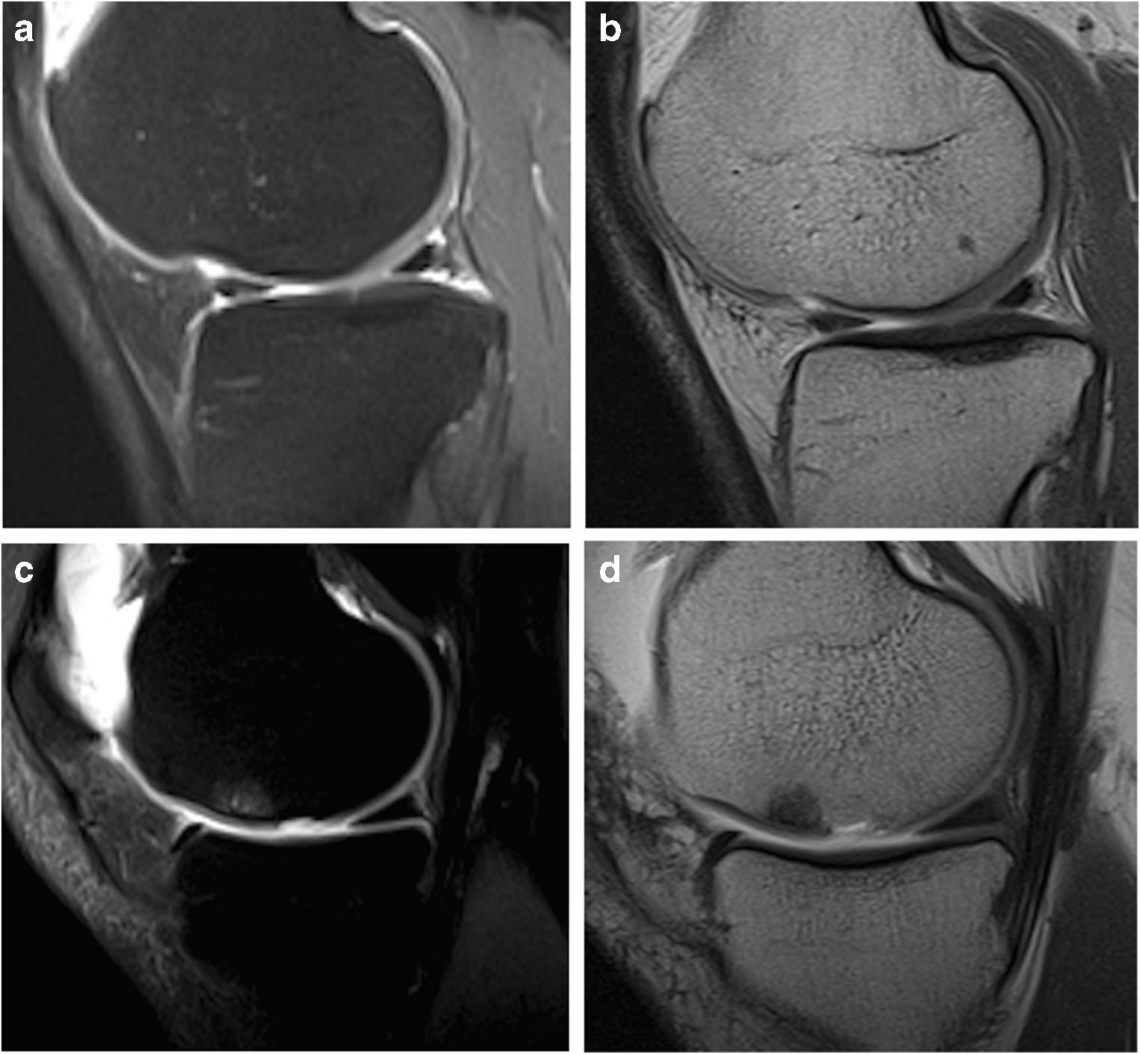
**a**, **b** A 28-year-old man treated with MACI of the internal tibial plate at long term with mMOCART score of 25, Cincinnati score 9. **a** Sagittal oblique FSE PD fat-saturated image shows structural incongruence of the chondral surface (arrowhead). **b** Sagittal oblique SE T1-weighted image. Contrast enhancement of the chondral implant (arrowhead). **c**, **d** A 47-year-old man treated with MACI of the internal femoral condyle at long-term with mMOCART score of 20, Cincinnati score 5. **c** Sagittal oblique FSE PD fat-saturated image shows structural incongruence of the chondral surface (arrow) and the presence of subchondral bone oedema with incomplete integration to the border zone. **d** Sagittal oblique SE T1-weighted image. Contrast enhancement of the chondral implant (arrow) [images obtained with “Unit1”]

Two of 26 patients totalized an mMOCART score of 25 points at mid-term while obtaining clinical scores of 9 and 10 (Fig. 6, Fig. 7); those patients maintained the dissociation between the two evaluation scales also at long term.

## Discussion

There are few studies with both clinical and radiological evaluation of MACI at 10-year follow up and, to our knowledge, the present study is the only one which performed MRI arthrography [8–19] to assess anatomical changes of MACI. Only in our previous experience in 2009, FU was obtained with MR arthrography [20].

Many of the analysed articles stop at an average of 5-year FU and only Aldrian et al. in 2014 [13] and Gille et al. in 2016 [19] evaluated the performance of MACI implants at long-term FU.

According to Kon et al., the reason for the lack of studies concerning long-term FU after MACI implants [8–11, 14, 15, 20] is due to the inherent difficulties encountered in following many patients on a decennial period.

We had an acceptable dropout rate of 30% given the long follow-up as many treated patients could not be reached or tracked; this is in accordance with the dropout rate in large studies of musculoskeletal disorders, ranged between 7 and 57% [22].

In our experience, at mid-term, the calculated mMOCART scores were satisfying, in accordance with the literature [8, 14, 15]: in 12/26 cases (46%), the scores were between 70 and 75 points and only in 4/26 cases (15%) the score was ≤35 points.

Those values were not confirmed by the subsequent long-term revaluation where only 2/26 patients (7%) scored between 70 and 75 points and almost half of them scored less than 35 points. The number of patients with complete coverage of the chondral defect, in particular, changed dramatically between mid-term and long term from 16/26 (61.5%) to 4/26 (15.3%).

The average mMOCART score had a statistically significant modification between mid-term and long-term FU (*p*=.00003) decreasing from 59.2 (SD ± 16.2) to 38.6 (SD ± 19.8).

Clinical scores had a slight worsening from an average score of 8.9 to an average score of 8.3. The difference was nonstatistically significant (*p*=.06).

The substantial stability of clinical scores is coherent with the results reported in the literature [18, 19, 23, 24], although this concordance must take into account the inhomogeneity of the clinical evaluation scales used.

In the present study, a statistically significant discordance between clinical and radiological scores was found.

The review of the literature regarding MRI and clinical controls at 5-year FU after MACI confirms a poor correlation between radiological and clinical data [8]. Even in our previous experience in 2011, the absence of a statistically significant correlation between these data was found [20]. This finding was also confirmed in the meta-analysis of De Windt et al. in 2013 including 1019 patients (FU variable from 6 months to 6 years), related to various cartilage surgery [25].

Carlovits et al., on the other hand, showed a progressive increase in both modified MOCART and clinical scores at 5-year FU on 21 MACI implants [10]. Gille et al. clinically evaluated 38 patients with 5 and 16 years FU: the results showed a significant increase in the final FU scores compared to the preoperative values (82.7 vs 59.6), with substantial stability if compared to the intermediate FU (82.7 vs 78.6) [19].

In the past years, only few articles based on the evaluation of MACI at the long-term FU (FU ≥ 10 years) were published [19, 26, 27].

Aldrian et al. in 2014 compared both clinical and radiological data after long-term FU in 16 patients who underwent MACI with a satisfactory 10-year average MOCART score (70.4) and a complete filling of the cartilage defect in 73% of cases; unfortunately, this study does not have a previous post-surgery MRI evaluation.

Clinical scores showed a steady improvement in the mean score up to 5 years. After 10 years, the scores decreased though remaining significantly higher than the preoperative values (59). A statistically significant correlation between radiological and clinical data was not found [13]. Subsequently, other studies based on the comparison between clinical and radiological findings confirmed those results [26, 27].

In our study, we demonstrated a statistically significant difference between the evolution of clinical and radiological parameters over time. In fact, the evolution of the mMOCART score, if compared to that of the Cincinnati score, demonstrated a significant reduction of the former with a non-significant reduction of the latter (Table 5).

This conclusion has an intrinsic limitation: the data obtained from the Cincinnati rating scale were not normally distributed like those of the mMOCART score. A non-parametric test had to be used in the first case while in the second case it was possible to analyse data with the Student *t* test for paired values. The evaluation of clinical data in the studies including FU ≥ 10 years showed an increase in the average long-term FU scores compared to the preoperative values [13, 19], which in our case were not available.

Our radiological results appear lower if compared to the studies with long-term FU: our average score (38.6) is, in fact, significantly lower than those reported in the literature (mean value 70.4). Furthermore, by analysing the degree of filling of the cartilage defect, in only 15% of patients at 10-year FU, a complete filling of the cartilage was found, disagreeing with Aldrian et al. (73%) [13].

To better understand our results, a clarification should be made: 12/40 patients (30%) who underwent MACI and participated at mid-term FU with MRa refused to submit to new clinical and radiological evaluation at long term, as they experienced subjective well-being.

Recently, Beck et al., with the histological evaluation on MACI implants, observed that all failed implants were characterized by fibrous involution independently from patients’ clinical conditions [28]. Those results may explain the poor correlation we observed between clinical and radiological scores at long-term FU.

As in our study the majority of the asymptomatic patients did not complete the radiological follow-up at long term (dropout 12/40), we suppose a possible correlation between the low mMOCART scores and a variable degree of fibrous involution of the implants.

Therefore, hypothetically, the fibrous degeneration could not be sufficient “per se” to cause significant clinical scores deterioration.

The study presented limitations: (1) the series is composed of only 26 patients; (2) it is mainly symptomatic patients since few of those in full well-being accepted the long-term check; (3) pre-intervention clinical evaluations were also not available.

In conclusion, MRI findings correlate poorly to clinical outcomes and should be viewed with caution.
